# IGF2‐Reprogrammed Macrophages Ameliorate the Inflammatory Response and Protect Against the Neuroinflammatory Process in Parkinson's Disease Models

**DOI:** 10.1111/acel.70020

**Published:** 2025-03-27

**Authors:** Felipe Grunenwald, Tomas J. Huerta, Denisse Sepulveda, Carolina Jerez, Valentina Urbina, Bárbara Carrera, Rodrigo Diaz‐Espinoza, Esteban Nova, Rodrigo Pacheco, Elisa Martín‐Montañez, Sara Gil‐Rodriguez, Nadia Valverde, María Garcia‐Fernandez, Carlos Aguilera, Pedro Chana‐Cuevas, René L. Vidal

**Affiliations:** ^1^ Center for Integrative Biology Universidad Mayor Santiago Chile; ^2^ Biomedical Neuroscience Institute University of Chile Santiago Chile; ^3^ Center for Geroscience, Brain Health and Metabolism Santiago Chile; ^4^ Departamento de Biología, Facultad de Química y Biología Universidad de Santiago de Chile Santiago Chile; ^5^ Departamento de Química, Facultad de Ciencias Naturales, Matemáticas y Medio Ambiente Universidad Tecnológica Metropolitana Santiago Chile; ^6^ Fundacion Ciencia & Vida Santiago Chile; ^7^ Centro Científico y Tecnológico de Excelencia Ciencia & Vida Fundación Ciencia & Vida Santiago Chile; ^8^ Facultad de Medicina y Ciencia Universidad San Sebastián Santiago Chile; ^9^ Departamento de Farmacología y Pediatría, Facultad de Medicina, Instituto de Investigación Biomédica de Málaga y Plataforma en Nanomedicina‐IBIMA Plataforma BIONAND University of Malaga Malaga Spain; ^10^ Departamento de Psicobiología y Metodología de las Ciencias del Comportamiento, Instituto de Investigación Biomédica de Málaga y Plataforma en Nanomedicina‐IBIMA Plataforma BIONAND University of Malaga Malaga Spain; ^11^ Department of Human Physiology, Faculty of Medicine, Biomedical Research Institute of Malaga, Faculty of Medicine University of Malaga Malaga Spain; ^12^ Hospital Fuerza Aérea de Chile Santiago Chile; ^13^ Centro de Trastornos del Movimiento (CETRAM), Facultad de Ciencias Médicas Universidad de Santiago Santiago Chile; ^14^ Escuela de Tecnología Médica Universidad Mayor Santiago Chile; ^15^ Escuela de Biotecnología Universidad Mayor Santiago Chile

**Keywords:** IGF2, macrophages, Parkinson's Disease, Therapy

## Abstract

Parkinson's disease (PD) is a neurodegenerative disorder characterized by the progressive loss of dopaminergic neurons in the Substantia Nigra, leading to motor impairment. A hallmark of PD is the presence of misfolded α‐synuclein (α‐syn) proteins and their neurotoxic accumulations, contributing to neuronal loss. Additionally, the inflammatory response plays a critical role in modulating the neurodegeneration process in PD. Moreover, peripheral macrophages recognize α‐syn, triggering chronic inflammation in both the bloodstream and brain tissue, leading to elevated levels of proinflammatory cytokines, as it was observed in PD patient samples. Insulin‐like growth factor 2 (IGF2) is a secreted factor with neuroprotective properties in several neurodegenerative disease models. Moreover, IGF2 signaling has been implicated in the cellular reprogramming of macrophages to an anti‐inflammatory phenotype through epigenetic changes. Recently, reduced IGF2 levels in both plasma and peripheral blood mononuclear cells (PBMCs) from PD patient samples were reported, suggesting a potential link between IGF2 levels and inflammation. In this study, we investigated the inflammatory profile of PD patients and the effect of IGF2‐reprogrammed macrophages in in vitro and in vivo PD models. Here, we report a significant increase in proinflammatory markers in PBMCs from PD patients. IGF2 treatment prevented α‐syn‐induced pro‐inflammatory profile in murine primary macrophages. Notably, IGF2‐reprogrammed macrophage treatment significantly reduced motor impairment, α‐syn accumulation, and microglial activation in the *Substantia Nigra* across different stages of disease progression in the PD preclinical model. These findings highlight the immunomodulatory effect of IGF2 on macrophages and its potential therapeutic impact on PD.

## Graphical abstract

Therapeutic Effects of IGF2‐Reprogrammed Macrophages (MIGF2) in Parkinson's Disease Progression in ASO Mice: This graphical abstract illustrates the therapeutic potential of IGF2‐reprogrammed macrophages (MIGF2) in mitigating Parkinson's disease progression in ASO mice. Bone marrow monocytes (BMM) treated with M‐CSF and recombinant IGF2 (rIGF2) generate MIGF2 macrophages, which, upon exposure to α‐synuclein pre‐formed fibrils (α‐syn PFF), exhibit reduced proinflammatory responses, diminished cytokine expression, and decreased TLR4 expression. Intravenous injection of MIGF2 in ASO mice leads to systemic immunomodulation and neuroprotective effects in the substantia nigra, including reduced microglial activation, decreased α‐synuclein accumulation, and improved motor function. A timeline of disease progression highlights systemic inflammation, neuroinflammation, dopaminergic neuronal loss, and motor symptoms over 3–23 months, reinforcing the role of MIGF2 in delaying neurodegeneration in Parkinson's disease.

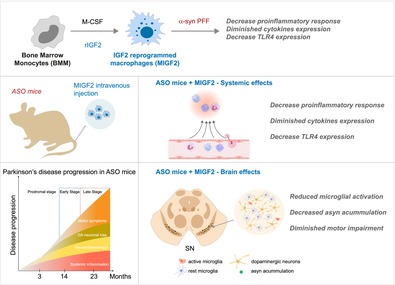

## 1 Introduction

Parkinson's disease (PD) is the second most prevalent late‐onset neurodegenerative disease and a leading cause of movement disorders (Forman et al. 2004; Vila and Przedborski 2004). Its incidence rises with age, affecting approximately 0.6% of individuals aged 65 to 69 and 2.6% of those aged 85 to 89 (de Lau and Breteler 2006). PD is characterized by the extensive loss of dopaminergic (DA) neurons in the *Substantia Nigra pars compacta* (SNpc) and the formation of protein inclusions called Lewy bodies, which are composed mainly of α‐synuclein (α‐syn) misfolded proteins and ubiquitinated proteins (Farrer 2006; Spillantini et al. 1997).

Although aging remains the primary known risk factor for developing idiopathic PD (de Lau and Breteler 2006), several epidemiological studies suggest that genetic and environmental factors also contribute to PD (Duvoisin and Johnson 1992; Muller‐Nedebock et al. 2023; Payami et al. 1994; Plante‐Bordeneuve et al. 1995). Moreover, recent advances in neuroimaging and post‐mortem neuropathology have led to a contemporary conceptualization of PD, proposing a disease subtype based on the site of onset of the pathogenic α‐syn (Borghammer et al. 2021; Horsager et al. 2020; Just et al. 2022). This includes a “body‐first subtype” where pathogenic α‐syn originates in the body and spreads to the brain, and a “brain‐first subtype” where pathogenic α‐syn originates in the brain and spreads to the body. This differentiation may have important implications for therapeutic intervention and disease management, as each could require a specific treatment strategy due to their distinct clinical manifestations and heterogeneity.

Currently, there is no cure for PD, and identifying treatments to delay or prevent PD symptoms is a major focus of the scientific community (Wolff et al. 2023). Inflammation has emerged as a key factor involved in the progression of the disease, strongly supported by studies on PD patients, where elevated levels of pro‐inflammatory cytokines are found in cerebrospinal fluid and brain tissue, indicating increased inflammation in the brain (Hirsch et al. 2012; Kouli et al. 2020; Mogi et al. 1996, 1994). Also, augmented pro‐inflammatory cytokine levels have been reported in both serum and peripheral blood mononuclear cells (PBMCs) of PD patients (Scalzo et al. 2010), showing a positive correlation with disease severity and rate of progression (Ahmadi Rastegar et al. 2019). Additionally, the activation of glial cells (microglia and astrocytes) and their role in PD pathology development have been widely reported (George et al. 2019; Gerhard et al. 2006; Hirsch et al. 2012; Knott et al. 1999). These findings suggest that understanding the role of systemic inflammation and neuroinflammation in PD progression could be a therapeutic approach to prevent or delay the neurodegeneration process.

Insulin‐like growth factor 2 (IGF2) is a secreted factor with neuroprotective properties in several models of neurodegenerative diseases, such as Alzheimer's disease (Pascual‐Lucas et al. 2014) and Huntington's disease (Garcia‐Huerta et al. 2020). Remarkably, genetic linkage studies have associated a polymorphism in the IGF2 gene with idiopathic PD (Sutherland et al. 2008). Additionally, we previously reported a significant decrease in IGF2 levels in both plasma and PBMCs in PD patients (Sepulveda et al. 2022) suggesting a role for IGF2 in PD pathology. Moreover, we and other investigators have described the neuroprotective effect of IGF2 in PD preclinical models, where IGF2 prevents dopaminergic neuronal death induced by α‐syn preformed fibrils, 1‐methyl‐4phenylpyridinium (MPP+) and 6‐hydroxydopamine (6‐OHDA), both in vitro (Romero‐Zerbo et al. 2025) and in‐vivo (Arcos et al. 2023; Martin‐Montanez et al. 2021; Zhang et al. 2023).

Recently, it has been shown that IGF2 signaling modulates the innate immune response of macrophages (Du et al. 2019; Wang et al. 2020). Low doses of IGF2 reprogram macrophages during their maturation, establishing an oxidative phosphorylation (OXPHOS) metabolism and promoting an anti‐inflammatory profile. This reprogramming is driven by the acquisition of distinct epigenetic marks, including changes in the deposition of the histone mark H3K27ac on inflammatory gene promoters (Du et al. 2019) and activation of the Glycogen synthase kinase‐3β (GSK3β)/ DNA (cytosine‐5)‐methyltransferase 3a (DNMT3a) axis (Wang et al. 2020). Moreover, several reports supported the beneficial use of IGF2‐reprogrammed macrophages in various inflammatory pathologies, including experimental autoimmune encephalomyelitis (EAE) (Du et al. 2019), dextran sodium sulfate (DSS)‐induced colitis (Chen et al. 2023), and liver cirrhosis (Yao et al. 2023); however, the use and possible impact of IGF2‐reprogrammed macrophages have not yet been reported in PD. In this study, we evaluate the inflammatory profile of PBMCs from PD patients and explore the effect of IGF2 on macrophages exposed to α‐syn oligomers, as well as the therapeutic impact of adoptive transfer cell therapy based on IGF2‐reprogrammed macrophages in PD preclinical models. We report an enhanced pro‐inflammatory profile in PBMCs from Chilean PD patients. Additionally, we observed that IGF2 suppresses inflammation in macrophages exposed to α‐syn oligomers. Notably, IGF2‐reprogrammed macrophages reduce motor impairment and systemic inflammation in a PD preclinical model at both symptomatic and presymptomatic stages. These results highlight the immunomodulatory role of IGF2 on macrophages, elucidating its beneficial impact as a cell therapy in PD.

## 2 Results

### 2.1 Systemic Inflammation Is Increased in PBMC From PD Patients

**FIGURE 1:**
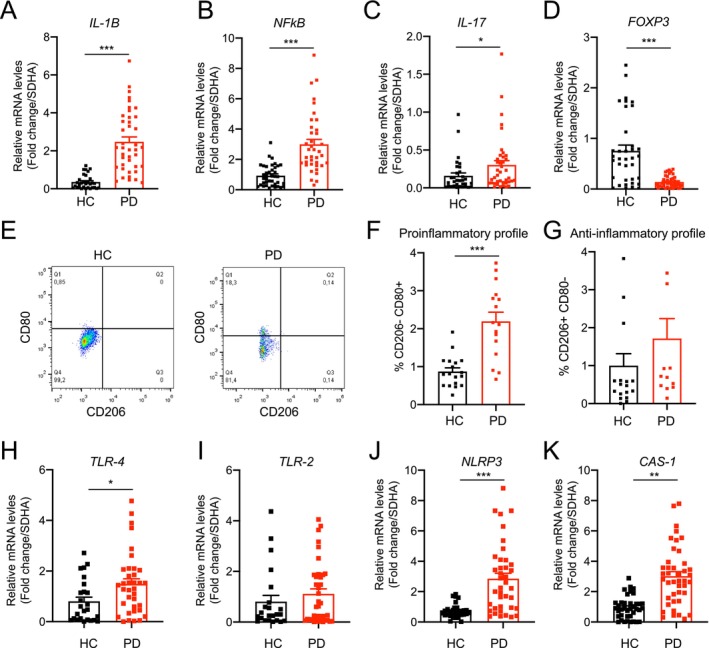
PD patients present an increase of proinflammatory cytokine and TLR4 levels in PBMCs. Total mRNA was extracted from freshly isolated peripheral blood mononuclear cells (PBMCs) from PD patients or health control (HC) subjects. (A) IL‐1B mRNA levels were quantified and normalized to SHDA levels. (B) NF‐κB mRNA levels were quantified and normalized to SHDA levels. (C) IL‐17 mRNA levels were quantified and normalized to SHDA levels. (D) FOXP3 mRNA levels were quantified and normalized to SHDA levels. (E) Flow cytometric analysis of PBMCs from healthy control (HC) subjects or PD patients. Cell selection parameters of macrophage cells (macrophage F4/80+ CD11b+) staining with surface marker CD206 and CD80. (F) Percentage of the macrophage CD206‐ CD80+ population. (G) Percentage of the macrophage CD206+ CD80‐ population. (H) TLR‐4 mRNA levels were quantified and normalized to SHDA levels. (I) TLR‐2 mRNA levels were quantified and normalized to SHDA levels. (J) NLRP3 mRNA levels were quantified and normalized to the SHDA levels. (K) Caspase‐1 (CAS‐1) mRNA levels were quantified and normalized to the SHDA levels. For all quantifications, statistically significant differences were detected using Mann–Whitney test (***p < 0.001; **p < 0.01; *p < 0.05). For all experiments, the mean and standard error are represented for PD (43 samples) and HC (40 samples).

PD is characterized by the selective dopaminergic neuronal loss and motor impairment symptoms, as well as systemic alteration, including changes in the immune system (Troncoso‐Escudero et al. 2018). To corroborate the increased immune response in Chilean PD patients, we analyzed inflammatory markers in PBMCs by qPCR. In our cohort, we observed a significant increase in the expression of pro‐inflammatory markers, including IL‐1B, Nuclear Factor kappa‐light‐chain‐enhancer of activated B cells (NF‐κB) and IL‐17 in PD patients compared with those healthy controls (HC) (Figure 1A–C). Conversely, we noted a decrease in the anti‐inflammatory marker Forkhead box P3 (FOXP3) in PD patients compared to HC (Figure 1D). Furthermore, we found that monocyte/macrophages derived from PD patients showed an enriched pro‐inflammatory population (CD80+/CD206−) compared with the HC group (Figure 1E,F), while the anti‐inflammatory population (CD80−/CD206+) remained unchanged (Figure 1G).

It has been described that α‐syn can be recognized by the family of Toll‐like receptors (TLR), primarily by TLR2 and TLR4, which are present in immune cells, including monocytes/macrophages (Campolo et al. 2019; Dzamko et al. 2017; Fellner et al. 2013; Hanamsagar et al. 2012). Therefore, we analyzed the gene expression of those TLR in PBMCs from PD patients. We found that PD patients showed a significant increase in the TLR4 expression levels (Figure 1H). In contrast, TLR2 expression levels were similar between the PD and HC groups (Figure 1I). Given the increase found in TLR4 receptor expression in PBMCs from PD patients, we evaluated NOD‐like receptor family pyrin domain containing 3 (NLRP3) and Caspase‐1 (CAS‐1) expression levels as a downstream of the TLR4 pathway. We observed a significant increase in the expression of both genes in PD patients (Figure 1J,K). These findings indicate an enhanced pro‐inflammatory profile in PBMCs from PD patients and suggest a role for the TLR4 pathway in the immune response.

### 2.2 Proinflammatory Response Triggered by α‐Syn Preformed Fibrils in Macrophages Was Prevented by IGF2 Reprogramming

As previously noted, peripheral monocytes/macrophages from PD patients exhibit an increase in the pro‐inflammatory population. Given that peripheral macrophages play a crucial role in regulating the inflammatory response by acquiring distinct inflammatory profiles in a stimulus‐dependent manner (Locati et al. 2020), including α‐syn oligomers (Sepulveda et al. 2022), we evaluate the effect of IGF2 treatment on the inflammatory response of macrophages exposed to α‐syn preformed fibrils (PFF).

Bone Marrow Monocytes (BMM) were isolated from the Wild‐Type (WT) and Thy‐1‐α‐synuclein overexpressing (ASO) mice and incubated with M‐CSF (20 ng/mL) to induce macrophage differentiation for 7 days, and flow cytometry was performed to determine the macrophage lineage. We observed an enriched population of macrophage‐positive cells in bone marrow samples from both WT and ASO mice, with no significant differences in the abundance of macrophage populations (Figure S1).

**FIGURE 2:**
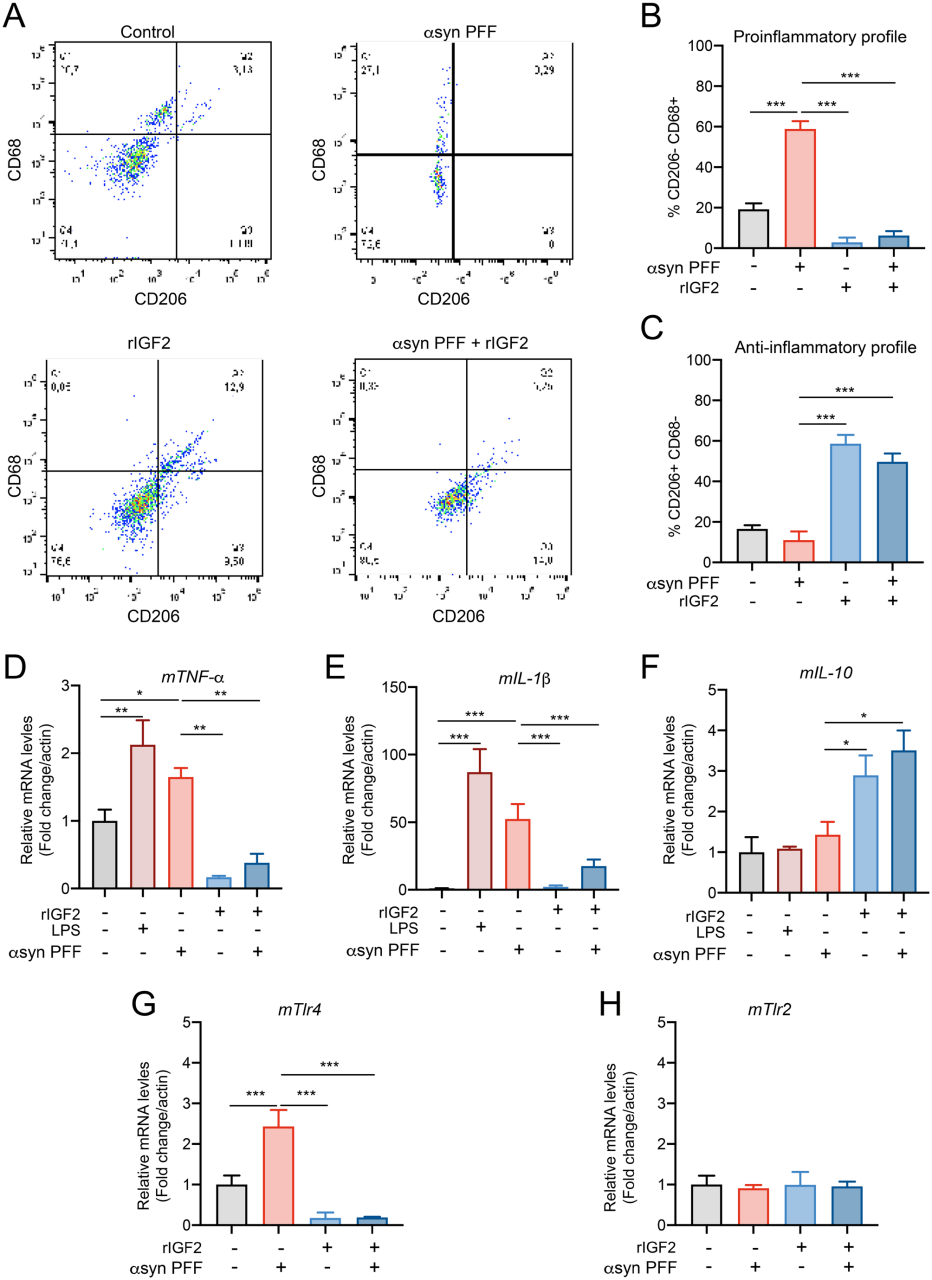
Proinflammatory response triggered by α‐syn preformed fibrils in macrophages was prevented by IGF2 treatment. Flow cytometric analysis of bone marrow monocytes under IGF2 pre‐treatment (5 ng/mL) and α‐syn preformed fibril stimulation (1ug/ml). (A) Cell selection parameters of macrophage cells (macrophage F4/80+ CD11b+) staining with surface markers CD206 and CD68. (B) Percentage of the macrophage CD206‐ CD68+ population. (C) Percentage of the macrophage CD206+ CD68‐ population. (D–H) Total mRNA extracts were generated from macrophage cultures from ASO mice reprogrammed with IGF2 and exposed to α‐syn PFF. (D) TNF‐α mRNA levels were quantified and normalized to β‐Actin levels. *E. IL* ‐1β mRNA levels were quantified and normalized to β‐Actin levels. (F) IL‐10 mRNA levels were quantified and normalized to β‐Actin levels. (G) TLR‐4 mRNA levels were quantified and normalized to β‐Actin levels. (H) TLR‐2 mRNA levels were quantified and normalized to β‐Actin levels. In all quantifications. statistically significant differences were detected one‐way ANOVA followed by Tukey's post‐test (***p < 0.001; **p < 0.01; *p < 0.05). For all experiments, the mean and standard error are represented for 5 samples per condition.

Previous reports indicate that IGF2 triggers an anti‐inflammatory profile in macrophages during their maturation (Du et al. 2019; Wang et al. 2020). Therefore, after isolating BMMs from WT and ASO mice, we treated them with recombinant mouse IGF2 (rIGF2) (5 ng/mL) for 7 days. Subsequently, IGF2‐reprogrammed macrophages were treated with 1 μg/mL of α‐syn preformed fibrils (PFF) and/or rIGF2 (5 ng/mL) for 24 h. We analyzed the cellular identity of macrophages by flow cytometry using CD68 and CD206, as a pro‐inflammatory marker and anti‐inflammatory marker, respectively. We observed a significant increase in the pro‐inflammatory response in macrophages exposed to α‐syn PFF. Remarkably, IGF2‐reprogrammed macrophages (MIGF2) showed a reduction in the pro‐inflammatory population following treatment with α‐syn PFF (Figure 2A,B). Also, IGF2 treatment enriched the anti‐inflammatory population, both in the absence and presence of α‐syn PFF (Figure 2A,C). Moreover, IGF2 treatment resulted in a decrease in the intracellular expression of inflammatory cytokines such as TNF‐α and IL‐1β (Figure 2D,E) and increased the intracellular expression of IL‐10 (Figure 2F) in macrophages exposed to α‐syn PFF. Next, we investigated whether the presence of α‐syn PFF affects TLR4 expression in macrophages. We found that macrophages treated with α‐syn PFF exhibited increased gene and protein expression levels of TLR4 compared to control cells (Figure 2G; Figure S2). Notably, this upregulation was prevented by IGF2 treatment (Figure 2G; Figure S2). We also evaluated TLR2 expression levels and found no changes in macrophages exposed to α‐syn PFF or treated with IGF2 (Figure 2H), consistent with observations in PBMCs from PD patients. These findings indicate an enhanced pro‐inflammatory profile in macrophages exposed to α‐syn PFF, which is prevented by IGF2 treatment.

### 2.3 Secretome of IGF2‐Reprogrammed Macrophages Reduced Neurotoxicity Induced by α‐Syn in an In Vitro PD Model

**FIGURE 3:**
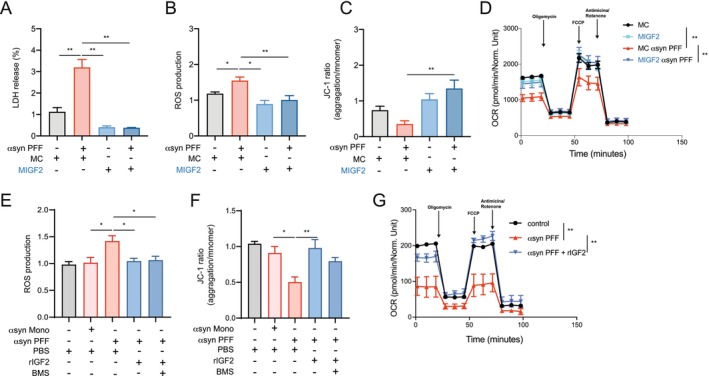
Secretome of MIGF2 or IGF2 prevents cytotoxicity and mitochondrial dysfunction in SN4741 cells exposed to α‐syn PFF. SN4741 were exposed to α‐syn PFF and conditional medium from macrophage reprogrammed with IGF2 (MIGF2) or conditional medium from macrophage treated with PBS (MC) for 24 h. Later, we determine cell viability and mitochondrial activity. (A) LDH cytotoxicity assay. (B) MitoSOX‐based flow cytometric assay used to detect mitochondrial ROS. (C) JC‐1 mitochondrial membrane potential assays. (D) Cell Mito Stress Test to measure the oxygen consumption rate (OCR) of live cells by mitochondrial respiration. SN4741 were exposed to α‐syn PFF and rIGF2 or PBS for 24 h. Later, we determine the mitochondrial activity. (E) MitoSOX‐based flow cytometric assay used to detect mitochondrial ROS. (F) JC‐1 mitochondrial membrane potential assays. (G) Cell Mito Stress Test to measure the oxygen consumption rate (OCR) of live cells by mitochondrial respiration. In all quantifications, statistically significant differences were detected one‐way ANOVA post‐test Tukey's (**p < 0.01; *p < 0.05). For all experiments, the mean and standard error are represented for 5 samples per condition.

As demonstrated earlier, IGF2 induces an anti‐inflammatory profile in macrophages exposed to α‐syn PFF, primarily by regulating the expression of pro‐inflammatory cytokines. To gain insight into the potential therapeutic impact of MIGF2 in PD, we treated SN4741 cells with MIGF2‐conditioned media or macrophages control (MC)‐conditioned media and evaluated cell viability. As expected, α‐syn PFF induced cell toxicity (Figure 3A), which was prevented by MIGF2‐conditioned media (Figure 3A) but not by the MC‐conditioned media.

Since dopaminergic neurons are particularly prone to reactive oxygen species (ROS) production (Basu et al. 2023; Trist et al. 2019) and because α‐syn has been reported to localize to mitochondria and contribute to the disruption of key mitochondrial processes, leading to an increased cellular vulnerability in dopaminergic neurons (Rocha et al. 2018), we evaluated mitochondrial function on the SN4741 cell line. As anticipated, α‐syn PFF triggered mitochondrial dysfunction in SN4741 cells, resulting in increased superoxide levels (Figure 3B) and a decrease in mitochondrial membrane potential, which was prevented by MIGF2‐conditioned media (Figure 3C). We also assessed the oxygen consumption rate (OCR) and observed a significant reduction in mitochondrial respiratory capacity induced by α‐syn PFF (Figure 3D). Notably, mitochondrial function was restored by the secretome of MIGF2 (Figure 3B–D).

Given that macrophages secrete IGF2 (Qiao et al. 2020) and it has been suggested that IGF2 modulates mitochondrial function (Wang et al. 2020; Zhou et al. 2023), we investigated whether IGF2 signaling prevents mitochondrial dysfunction triggered by α‐syn PFF in SN4741 cells. Interestingly, IGF2 treatment restored mitochondrial function in SN4147 cells exposed to α‐syn PFF (Figure 3F–H), similar to what was observed with the MIGF2‐conditional media. Moreover, BMS‐754807 (BMS), a potent inhibitor of the IGF‐1R/IR family kinases (Carboni et al. 2009), did not alter the neuroprotective effect of rIGF2 (Figure 3F–H), suggesting that this phenomenon is dependent on IGF2 receptor (IGF2R) signaling. Taken together, these results indicate that the secretome of MIGF2 exerts a neuroprotective effect in the PD cellular model, potentially through the secretion of IGF2.

### 2.4 IGF2‐Reprogramated Macrophages Treatment Reduced Motor Impairment and the Inflammatory Response in a Preclinical PD Model

As shown earlier, IGF2 treatment modifies the response of macrophages exposed to α‐syn PFF and triggers an anti‐inflammatory profile that has a neuroprotective effect in the PD cellular model. To explore the possible therapeutic outcome of these IGF2‐reprogrammed macrophages in PD, we administered those cells to ASO mice, as a PD preclinical model in different stages of disease progression.

**FIGURE 4:**
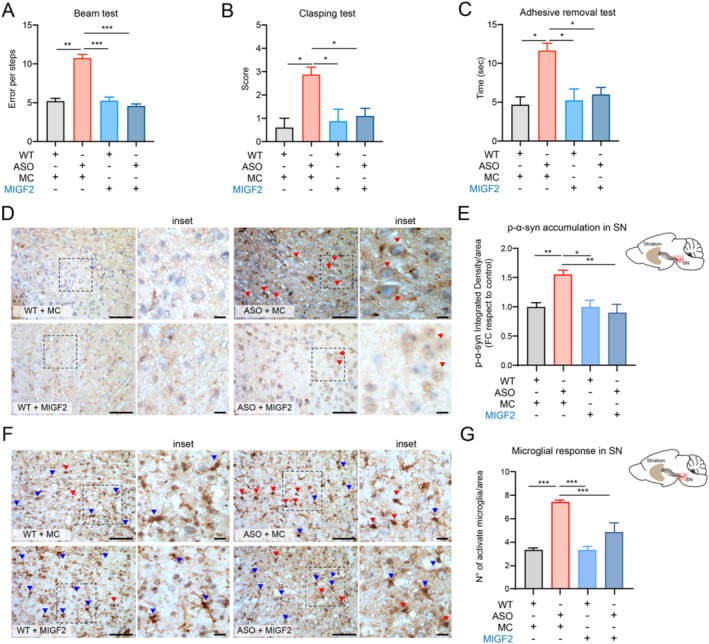
IGF2‐reprogrammed macrophages decrease motor impairment, α‐syn accumulation and microglial activation in preclinical PD model at 23 months. Motor performance was evaluated in 23‐month‐old ASO or WT mice with macrophages reprogrammed with IGF2 (MIGF2) or macrophages reprogrammed with PBS (MC). (A) The number of errors for crossing the beam was quantified in each condition. (B) Score of the Clasping test was quantified for 3 min in each condition. (C) The time spent on adhesive removal from the nose was quantified in each condition. Immunohistochemistry was performed on the sagittal section of brain tissue from 23‐month‐old ASO, or WT mice treated with macrophages reprogrammed with IGF2 (MIGF2) or macrophages reprogrammed with PBS (MC). (D) p‐α‐syn immunodetection in the sagittal section from ASO or WT treated with MC or MIGF2. p‐α‐syn are represented with red arrows. (E) Determination of integrity density of p‐α‐syn in SN brain region. (F) IBA1 immunodetection in SN region from ASO or WT treated with MC or MIGF2. The active microglia cells are represented in red arrows and resting microglia cells are represented in blue arrows. (G) Quantification of the number (N°) of activate microglia in SN brain region. Scale bar 50 μm for panels D and F and 20 μm for inset. In all quantifications, statistically significant differences were detected one‐way ANOVA post‐test Tukey's (***p < 0.001; **p < 0.01; *p < 0.05). For all experiments, the mean and standard error are represented 5 mice per condition.

PD manifestation increases during aging and is considered a significant factor in triggering clinical motor symptoms. A similar phenomenon has been reported in ASO mice (Chesselet et al. 2012; Richter et al. 2023). For this reason, we wondered whether MIGF2 could have a neuroprotective effect at a later stage of disease. We intravenously inject 23‐month‐old ASO mice with MIGF2 or MC once a week for 1 month, followed by an analysis of motor performance. As expected, 23‐month‐old ASO mice exhibited impaired motor performance, and surprisingly, MIGF2 treatment significantly decreased motor impairment (Figure 4A–C). As previously reported, the α‐syn accumulation in the brain tissue increases during disease progression, which positively correlates with motor deterioration in ASO mice (Richter et al. 2023). Therefore, we evaluated α‐syn accumulation by the detection of phosphor‐α‐syn (p‐α‐syn) in the *Substancia Nigra* (SN) brain region of 23‐month‐old ASO mice. Remarkably, we observe that MIGF2 treatment triggers a reduction of p‐α‐syn accumulation (Figure 4D,E). Additionally, we evaluated the total α‐syn levels by Western Blot analysis, noting a significant increase in α‐syn levels in ASO mice compared to WT mice, with a tendency to decrease with MIGF2 treatment (Figure S3). Furthermore, ASO mice display enhanced microgliosis, which impacts neuronal survival (Richter et al. 2023). We analyzed the microglial activation in the SN brain region by evaluating the number and morphology as described previously (Jin and Yamashita 2016; Kreutzberg 1996; Wittekindt et al. 2022), and we determined that MIGF2 treatment decreases the number of microglial activation in the Substancia nigra brain region of ASO mice compared with the control group (Figure 4F,G).

**FIGURE 5:**
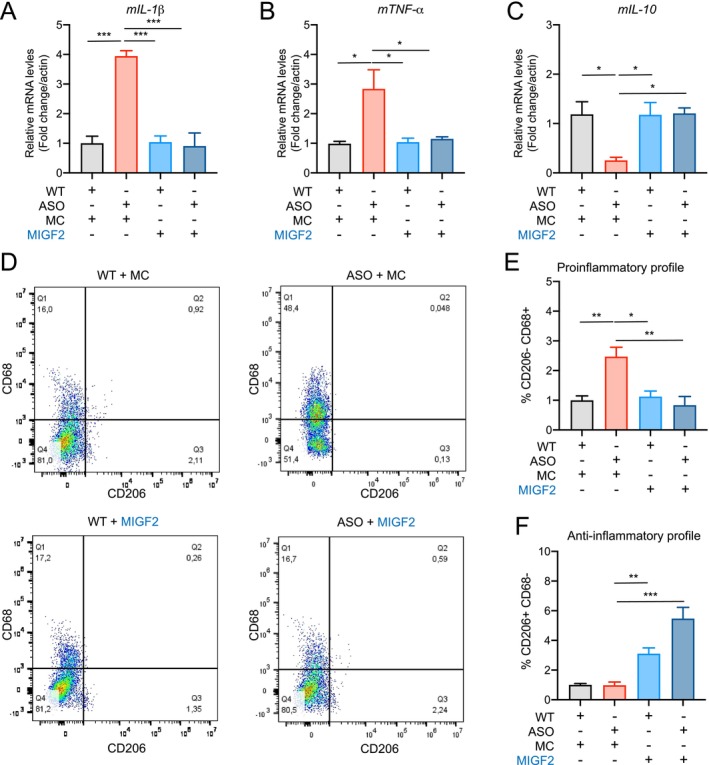
IGF2‐Reprogrammed macrophages reduce systemic inflammation in preclinical PD model at 23 months. (A–C) Total mRNA was extracted from freshly isolated peripheral blood mononuclear cells (PBMCs) from the ASO, and WT mice treated. (A) mIL‐1β mRNA levels were quantified and normalized to β‐Actin levels. (B) mRNA levels of mTNF‐α were quantified and normalized to β‐Actin levels. (C) mIL‐10 mRNA levels were quantified and normalized to β‐Actin levels. (D–F) Flow cytometric analysis of isolated peripheral blood mononuclear cells (PBMCs) from 23‐month‐old ASO or WT mice treated with MIGF2 or MC. (D) Cell selection parameters of macrophage cells (macrophage F4/80+ CD11b+) staining with surface marker CD206 and CD68. (E) Percentages of CD206‐/CD68+ macrophages population. (F) Percentages of CD206+/CD68‐ macrophages population. In all quantifications, statistically significant differences were detected through one‐way ANOVA post‐test Tukey's (***p < 0.001; **p < 0.01; *p < 0.05). For all experiments, the mean and standard error are represented 5 mice per condition.

As mentioned before, PD patients exhibit increased systemic inflammation, which is also observed in ASO mice (Richter et al. 2023). Considering the peripheral administration of the treatment, we evaluated the effect of MIGF2 on systemic inflammation. We detected a significant decrease in IL‐1β levels and an increase in IL‐10 levels in PBMCs from ASO mice treated with MIGF2 (Figure 5A–C). Furthermore, considering the results obtained in PBMCs from PD patients, we analyzed the TLR4 signaling pathway by measuring NLRP3 and CAS‐1 expression levels. We observed a significant increase in the expression of these genes in ASO mice compared to the WT mice; however, only CAS‐1 showed a significant decrease following MIGF2 treatment (Figure S4A,B). Moreover, when examining the inflammatory profile of PBMCs from ASO mice, we noted that MIGF2 treatment significantly reduced the pro‐inflammatory macrophage population in 23‐month‐old ASO mice (Figure 5D,E). However, we did not observe significant changes in the anti‐inflammatory macrophage population in any of the experimental groups (Figure 5D,F).

**FIGURE 6:**
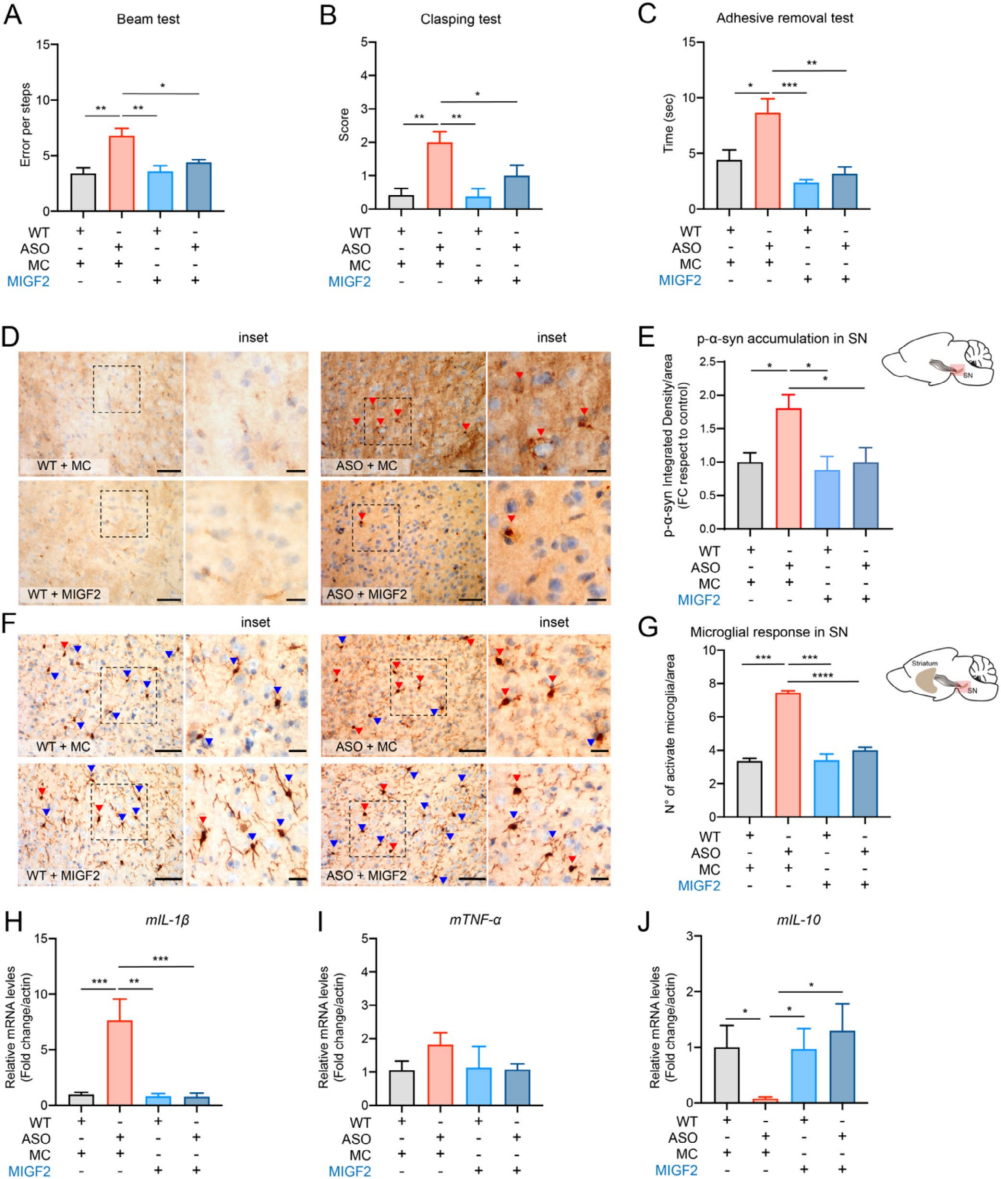
IGF2‐Reprogrammed macrophages reduce motor impairment, α‐syn accumulation, microglia activation and systemic inflammation at 14 months in ASO mice. Motor performance was evaluated in 14‐month‐old ASO or WT mice with macrophages reprogrammed with IGF2 (MIGF2), or macrophages reprogrammed with PBS (MC). (A) The number of errors for crossing the beam was quantified in each condition. (B) Score of the Clasping test was quantified for 3 min in each condition. (C) The time spent on adhesive removal from the nose was quantified in each condition. (D, E) Immunohistochemistry was performed on the sagittal section of brain tissue from 14‐month‐old ASO, or WT mice treated with macrophages reprogrammed with IGF2 (MIGF2) or macrophages reprogrammed with PBS (MC). (D) p‐α‐syn immunodetection in the sagittal section from ASO or WT treated with MC or MIGF2. p‐α‐syn are represented with red arrows. (E) Determination of integrity density of p‐α‐syn in SN brain region. (F) IBA1 immunodetection in SN region from ASO or WT treated with MC or MIGF2. The active microglia cells are represented in red arrows and resting microglia cells are represented in blue arrows. (G) Quantification of the number (N°) of activate microglia in SN brain region. Scale bar 50 μm for panels D and F and 20 μm for inset. (H–J) Total mRNA was extracted from freshly isolated peripheral blood mononuclear cells (PBMCs) from the ASO and WT mice treated. (H) mIL‐1β mRNA levels were quantified and normalized to β‐Actin levels. (I) mRNA levels of mTNF‐α were quantified and normalized to β‐Actin levels. (J) mIL‐10 mRNA levels were quantified and normalized to β‐Actin levels. In all quantifications, statistically significant differences were detected one‐way ANOVA post‐test Tukey's (***p < 0.001; **p < 0.01; *p < 0.05). For all experiments, the mean and standard error are represented for 7–8 mice per condition.

As widely reported, neuronal dysfunction and degeneration associated with PD begins long before the symptoms manifest (Simon et al. 2020). ASO mice, a progressive model of PD, typically exhibit the onset of motor symptoms at 14 months of age. We investigated whether MIGF2 treatment could provide benefits at the early stages of the disease to prevent the development of motor symptoms. Following the same therapeutic strategy in 14‐month‐old ASO mice, we observed the expected impaired motor performance at this age. Notably, MIGF2 treatment reduced motor impairment (Figure 6A–C). It has been described that prior to the onset of motor symptoms, there is an α‐syn accumulation, which further impacts DA neuron function (Richter et al. 2023). We evaluated α‐syn accumulation in 14‐month‐old ASO mice and observed that MIGF2 treatment led to a decrease in p‐α‐syn accumulation in the SN brain region (Figure 6D,E). Additionally, we noted a reduction in microglial activation in MIGF2‐treated ASO mice compared to those treated with MC (Figure 6F,G). Since we observed that MIGF2 treatment also modifies the systemic inflammation in 23‐month‐old ASO mice, we speculate that a similar outcome occurs at an earlier stage of the disease. We assessed the effect of MIGF2 on systemic inflammation, and we observed a significant decrease in IL‐1β levels and an increase in the IL‐10 levels in PBMCs from ASO mice treated with MIGF2 (Figure 6H–J). Additionally, we analyzed the TLR4 signaling pathway by measuring NLRP3 and CAS‐1. We noted a significant increase in the expression of NLPR3 in ASO mice compared to the controls; however, no significant differences were observed following MIGF2 treatment (Figure S4C,D). Moreover, MIGF2 treatment showed a significant reduction in the macrophage pro‐inflammatory population in 14‐month‐old ASO mice (Figure S5A,C). However, we did not observe significant changes in the macrophages anti‐inflammatory population in either of the experimental groups (Figure S5B,C).

Considering that in ASO mice the pathology starts with changing the systemic and neuroinflammation as early as 2monthsold (Richter et al. 2023), We decided to evaluate whether MIGF2 treatment could prevent further inflammation and disease progression. In 3‐month‐old ASO mice, we observed an increase in IL‐1β and TNF‐α levels (Figure S6A,B), which were reduced by MIGF2 treatment (Figure S6A,B). However, IL‐10 levels remained unchanged by MIGF2 treatment (Figure S6C). Additionally, the proinflammatory and anti‐inflammatory macrophage populations were similar in WT and ASO mice (Figure S6D–F), and MIGF2 treatment did not affect the macrophage inflammatory profile (Figure S6D–F).

Taken together, these results show the efficacy of MIGF2 treatment in ameliorating the PD symptoms at different stages of the disease.

## 3 Discussion

It has been demonstrated that PD is characterized not only by motor symptoms but also by non‐motor symptoms associated with gut inflammation, REM sleep alteration, and an increased inflammatory response (Elgueta et al. 2019; Joers et al. 2020; Ramanzini et al. 2022; Schapira et al. 2017; Wang et al. 2015). In our study, we found a significant upregulation in the expression of pro‐inflammatory markers (IL‐1B, IL‐17, and NF‐κB) and an increase in the pro‐inflammatory population of monocyte/macrophages in blood samples from Chilean PD patients. This further corroborates the involvement of systemic inflammation in PD, as it was seen in other PD patient cohorts (Lin et al. 2019; Smajic et al. 2022; Starhof et al. 2018; Storelli et al. 2019). To our knowledge, this is the first report that shows an increase in the pro‐inflammatory response in PBMCs from Chilean and/or Latin‐American PD patients, suggesting that further therapies could be focused on ameliorating the inflammatory response and therefore improve DA neuronal survival.

It has been hypothesized that α‐syn in the bloodstream plays a role in activating the immune system (Basu et al. 2023) through various receptors, including TLRs, particularly TLR‐2 and TLR‐4 (Gorecki et al. 2021; Heidari et al. 2022; Li et al. 2021). In line with these reports, our results showed an increased TLR4 expression in PBMCs derived from PD patients, with no changes in TLR2 levels. This finding is consistent with the increased expression of NF‐kB, CAS‐1, NLRP3, and IL‐1β, all of which are downstream targets of the TLR4 signaling pathway. These results highlight the crucial role of TLR4 in PD, as it is involved in the recognition of extracellular α‐syn and, therefore, the initiation of the pro‐inflammatory process (Gorecki et al. 2021; Heidari et al. 2022; Li et al. 2021).

IGF2 is a secreted factor that plays a different role in metabolism, proliferation, and cellular differentiation, and it has a neuroprotective effect in different neurodegenerative diseases, including PD (Alberini 2023; Troncoso‐Escudero et al. 2020). Recently, it has been reported to play a crucial role in immune homeostasis and inflammatory response (Du et al. 2019; Guo et al. 2023; Yao et al. 2023; Zhang et al. 2020), particularly in macrophages, by promoting the acquisition of an anti‐inflammatory phenotype during maturation (Du et al. 2019). Notably, we previously reported that in PD patients, decreases in IGF2 occur both in plasma and PBMCs, with no changes in IGF1 levels (Sepulveda et al. 2022). This decrease may relate to the increased pro‐inflammatory profile observed in PBMCs demonstrated by this study.

As proof of concept of this hypothesis, mouse bone marrow‐derived macrophages (BMDMs) reprogrammed with IGF2 and exposed to α‐syn PFF showed a decrease in the inflammatory response, evidenced by a decrease in the expression of IL‐1β, TNF‐α, TLR4, CD68, and MHCII. More surprisingly, these macrophages also acquire an anti‐inflammatory profile, characterized by increased expression of IL‐10 and CD206, further supporting the notion that IGF2 regulates the inflammatory response in macrophages, as it was suggested previously (Du et al. 2019; Wang et al. 2020).

A major limitation of this study is unraveling the molecular mechanism underlying the anti‐inflammatory profile triggered by IGF2 in macrophages exposed to α‐syn PFF. It has been described that IGF2, through IGF2R, induces epigenetic remodeling by decreasing the expression of pro‐inflammatory genes while also reducing the phosphorylation of RAC (Rho family)‐alpha serine/threonine‐protein kinase (AKT/PKB)/Mammalian target of rapamycin (mTOR) upon LPS stimulation, preventing the switch‐off of OXPHOS and promoting the initiation of autophagy (Du et al. 2019). In fact, it has been described that some autophagy‐related genes, including Beclin‐1 and Autophagy‐related protein 5 (ATG 5), are upregulated in macrophages exposed to α‐syn and IGF2 (Sepulveda et al. 2022), suggesting that the acquisition of an anti‐inflammatory profile in macrophages by IGF2 involves autophagy regulation, which could facilitate the degradation of misfolded α‐syn (Haenseler et al. 2017). Moreover, it has been shown that upon IGF2R activation by IGF2, it translocates to the nucleus and recruits GSK3β which in turn activates DNMT3a/b, altering the methylation landscape and triggering an anti‐inflammatory profile (Wang et al. 2020). Interestingly, AKT is an inhibitor of GSK3β suggesting that IGF2/IGF2R activates GSK3β but also decreases its inhibition (Jere et al. 2019). Given that LPS and α‐syn could be recognized both by TLR4 (Heidari et al. 2022), it is possible that a similar molecular mechanism is involved in triggering the anti‐inflammatory response observed in this study; however, this hypothesis needs to be proven. An alternative molecular mechanism is associated with the regulation of anti‐inflammatory‐related genes. Recently, it was observed that lower doses of IGF2 increase the expression of anti‐inflammatory genes, including CD163, CD206, and Nuclear Receptor Subfamily 4 Groups A Member 2 (NR4A2)/Nurr1 genes (Yao et al. 2023). NR4A2/Nurr1 has been associated with triggering an anti‐inflammatory response in macrophages (Mahajan et al. 2015; Xiong et al. 2024; Yao et al. 2023). Furthermore, NR4A2/Nurr1 plays a crucial role in suppressing the pro‐inflammatory response upon activation of the TLR4/NFkB pathway by LPS and α‐syn (Shao et al. 2019). Remarkably, our results showed that IGF2 decreases the TLR4 expression in macrophages while also increasing the expression of anti‐inflammatory molecules, including CD206 and IL‐10, suggesting that the anti‐inflammatory profile triggered in IGF2‐reprogrammed macrophages exposed to α‐syn could be associated with increased activation of NR4A2/Nurr1; however, this molecular mechanism remains to be elucidated.

ASO mice are widely used to study PD, as they recapitulate key aspects of the disease, including progressive motor impairment, neurodegeneration, inflammation, and α‐syn misfolded aggregation (Chesselet et al. 2012). Our findings show that MIGF2 administration, compared to MC, alleviates motor deficits, reduces α‐syn accumulation, and mitigates microglia activation in the symptomatic stage of PD in ASO mice. Moreover, MIGF2 treatment reduced systemic inflammation at multiple disease stages by decreasing pro‐inflammatory cytokines expression in PBMCs, reflecting beneficial effects similar to those observed in other inflammatory conditions, such as dextran sodium sulfate (DSS)‐induced colitis (Chen et al. 2023) and liver cirrhosis (Yao et al. 2023). This evidence underscores the relevance of restoring inflammatory homeostasis to alleviate motor symptoms in this α‐syn‐based PD preclinical model.

Notably, analysis of brain tissue from MIGF2‐treated ASO mice revealed reduced microglial activation and α‐syn accumulation, implying that the MIGF2 effect extends beyond peripheral inflammation and directly affects the brain. Nevertheless, the mechanism by which MIGF2 exerts its neuroprotective effect remains elusive. It has been suggested that macrophages migrate and infiltrate the tissue to regulate the inflammatory response, even having the ability to cross the blood–brain barrier (Winkler et al. 2021). This raises the possibility that MIGF2 exerts therapeutic and neuroprotective effects in brain tissue by secreting anti‐inflammatory factors, including IGF2 (Qiao et al. 2020), which has been shown in cancer‐related studies to act as an IGF2arrier (Liang et al. 2021). Therefore, IGF2 may exert direct neuroprotective effects, as previous studies have demonstrated its role in preventing dopaminergic neuronal death in in vitro and in vivo PD models (Romero‐Zerbo et al. 2025). In our in vitro experiments, we found that the MIGF2 secretome–rather than the MC secretome–prevents α‐syn oligomer‐induced neurotoxicity and mitochondrial dysfunction in SN4741 cells, indicating its role in promoting DA neuronal survival. As proof of concept for MIG2‐induced IGF2 secretion, IGF2 treatment also prevents α‐syn oligomer‐induced neurotoxicity and mitochondrial dysfunction, further confirming IGF2's neuroprotective role in PD, as demonstrated by our laboratory and others; but more importantly, this suggests that MIGF2's beneficial effects may be mediated through IGF2 secretion. Future studies should investigate MIGF2's capacity to migrate to brain tissue and whether the neuroprotective effect is directly on DA neurons or indirectly by modulating glial cells, such as microglia.

Current therapies for PD improve motor impairment but do not prevent further neurodegeneration and α‐syn misfolded aggregation, leading to diminished efficacy as the disease progresses (Wolff et al. 2023). Remarkably, our study demonstrates that MIGF2 treatment reduces both systemic and brain inflammation at an earlier and later symptomatic stage in ASO mice (3, 14, and 23‐month‐old) accompanied by decreased motor impairment and α‐syn accumulation in the brain. This finding suggests that MIGF2 treatment may be effective even in symptomatic and advanced stages of PD, where DA neuron degeneration is more severe (Tolosa et al. 2021). This expands the potential clinical applications of MIGF2 therapy in PD. Further studies should focus on determining the duration of MIGF2 efficacy; however, based on similar therapeutic strategies, the treatment may remain effective for several months after the last injection, particularly if macrophages infiltrate the host tissue (Wroblewska et al. 2023).

Our findings highlight the potential of IGF2 as an immunomodulatory molecule in PD and underscore the need to investigate the molecular mechanism underlying IGF2 signaling on macrophages exposed to α‐syn PFF in vitro and how IGF2‐reprogrammed macrophages exert their beneficial effects on ASO mice, which is fundamental to developing this novel therapeutic approach for PD.

## 4 Experimental Procedures

### 4.1 Study Participants and Clinical Evaluation

These experiments were performed in the Neurology department of Hospital Clínico Fuerza Aérea de Chile (FACH) and processed in the Center for Integrative Biology (CIB) of Universidad Mayor in Santiago, Chile, in accordance with the Declaration of Helsinki. The local ethical committee of the FACH hospital and Mayor University approved them.

All the participants, 43 PD patients and 41 healthy controls, were recruited and signed written informed consent. PD patients were evaluated with clinical history, physical examination, and received a score from the Hoehn and Yahr modified (Goetz et al. 2004) and UPDRS (Movement Disorder Society Task Force on Rating Scales for Parkinson's Disease 2003; Palmer et al. 2010). We considered as inclusion criteria subjects aged over 18 years, having a history of attention in Hospital FACH, subjects with a diagnosis of PD in Stage 1–4 of Hoehn and Yahr modified scale, and signed informed consent. On the other hand, exclusion criteria were age under 18 years and subjects diagnosed with PD in Stage 5 of Hoehn and Yahr modified scale.

### 4.2 Plasma and PBMCs Isolation

The blood sample was 30 mL of venous blood extracted from the FACH Hospital and Centro de Trastornos del Movimiento (CETRAM) in an EDTA‐Purple top blood collection tube.

Plasma and peripheral blood mononuclear cell (PBMCs) fractions from humans and mice were obtained based on centrifugation and usingFicoll protocols (Biddison 2001; Garcia‐Huerta et al. 2020). The EDTA‐treated blood will be placed gently over Ficoll (1:1 ratio; 17,144,002, Cytiva) and then centrifuged at 4000×*g* for 30 min in an unbraked centrifuge. The ring of cells (PBMCs) obtained in the interface of the Ficoll, and the serum will be recovered and washed twice with sterile phosphate‐buffered saline (PBS). Then, the PBMCs enriched fraction will be separated for RNA isolation and flow cytometry analysis.

### 4.3 Preparation of Bone Marrow‐Derived Macrophages (BMDMs)

Thy1‐α‐synuclein overexpressing (ASO) mice at 3 months old (Rockenstein et al. 2002) were euthanized by isoflurane overdose. Bone marrows from the femur cavity were extracted as previously described (Zhang et al. 2008). Briefly, bone marrows were extracted with a needle using a sterile 15‐mL tube. Cells were centrifuged for 5 min at 500×*g*, at room temperature. Cell pellets containing macrophages were resuspended with complete medium, DMEM/F12 (10569‐010, Gibco, ThermoFischer Scientific) supplemented with 10% Fetal bovine serum (FBS), 1% penicillin/streptomycin, 20 ng/mL macrophage colony‐stimulating factor (MCS‐F) (PHC‐9501, ThermoFischer Scientific), by tapping the tube, pipetting up and down, then transferring them to a 100 mm sterile plate. Macrophages were maintained at 37°C, 5% CO_2_ incubator for 7 days. To determine the effect of IGF2 on the inflammatory profile, macrophages were treated with recombinant mouse IGF2 (5 ng/mL, 792‐MG, R&D Systems) for 7 days (Du et al. 2019). After IGF2 treatment, macrophages were stimulated with 1 μg/mL of recombinant mouse α‐synuclein PFFs (α‐syn PFFs, AG938, Sigma) or PBS for 24 h. α‐syn PFFs were generated as previously described (Mahul‐Mellier et al. 2015).

### 4.4 RNA Extraction From PBMCs or Primary Macrophages

RNA extraction and cDNA synthesis from PBMCs (humans and mice) and macrophages was performed to evaluate the inflammatory‐related mRNA levels by real‐time PCR.

The human primers used were: IL‐1 beta (IL‐1B) FW (AAA CAG ATG AAG TGC TCC TTC CAG G), RV (TGG AGA ACA CCA CTT GTT GCT CCA); NF‐kB FW (ATG GCT TCT ATG AGG CTG AG), RV (GTT GTT GTT GGT TCG GAT GC); Foxp3 FW (CAA GTT CCA CAA CAT GCG AC), RV (ATT GAG TGT CCG CTG CTT CT); TLR‐4 FW (TTT ATT CAG AGC CGT TGG TG), RV (CAG AGG ATT GTC CTC CCA TT); TLR‐2 FW (CAG CTG GAG AAC TCT GAC CC), RV (CAA AGA GCC TGA AGT GGG AG); NLRP3 FW (GAT CTT CGC TGC GAT CAA CA) NLRP3 RV (GGG ATT CGA AAC ACG TGC ATTA); Caspase‐1 (CAS‐1) FW (GCC TGT TCC TGT GAT GTG GAG) CAS‐1 RV (TGC CCA CAG ACA TTC ATA CAG TTT C); IL17 FW (ACT CCT GGG AAG ACC TCA TTG), RV (GGC CAC ATG GTG CAC AAT CG); SHDA FW (GAG GCA GGG TTT AAT ACA GCA), RV (CCA GTT GTC CTC CTC CAT GT).

Murine primers used were: TNF‐alpha (TNF‐α) FW (ATG AAC GCT ACA CAC TGC ATG), RV (CCA TCC TTT TGC CAG TTC CTC); IL‐1 beta (IL‐1β) FW (AAA CAG ATG AAG TGC TCC TTC CAG G), RV (TGG AGA ACA CCA CTT GTT GCT CCA); IL10 FW (AAG GCA GTG GAG CAG GTG AA), RV (CCA GCA GAC TCA ATA CAC AC); TLR4 FW (CAG AGG ATT GTC CTC CCA TT), RV (TTT ATT CAG AGC CGT TGG TG); TLR2 FW (CAG CTG GAG AAC TCT GAC CC), RV (CAA AGA GCC TGA AGT GGG AG); β‐actin FW (CCA GTT GGT AAC AAT GCC ATG T), RV (GGC TGT ATT CCC CTC CAT CG).

### 4.5 Flow Cytometry of PBMCs

Human and mice PBMCs samples were resuspended in 300 μL of PBS1x and incubated with primary antibodies for CD45, CD11b, CD14, F4/80, CD80, and CD206 for 30 min, followed by staining with Zombie aqua (1:1000, 77,143, Biolegend) for an additional 15 min. Subsequently, samples were fixed with 2% paraformaldehyde (PFA) for 5 min and washed with PBS1x. Flow cytometry was performed to determine macrophages' inflammatory profile by CytoFLEX cytometer (A00‐1‐1102, Beckman Coulter) and the data were analyzed with FlowJo software.

The macrophage profile from Human PBMC samples was assessed by selecting single cells, followed by live/dead selection with Zombie aqua. Lymphoid lineage was identified by CD45 (1:1000, 3,685,509, PE, BioLegend), and macrophage cell types were selected using CD11b (1:1000, 101,261, APC/Fire 750, BioLegend) and CD14 (1:1000, 367,117, APC, BioLegend). The inflammatory profile was evaluated with CD80 (1:1000, 305,213, Alexa Fluor 488, BioLegend) and CD206 (1:1000, 321,125, Brilliant violet 421, BioLegend), for the pro‐inflammatory and anti‐inflammatory identities, respectively.

In WT and ASO mice PMBCs samples, macrophage profiling was performed by using single‐cell selection; then, live/dead selection was conducted using Zombie aqua. Lymphoid lineage was identified by CD45 (1:1000, 562,420, PE‐CF594, BD). Macrophage cell type was selected with CD11b (1:1000, 101,206, FITC, BioLegend) and F4/80 (1:1000, 566,787, APC, BD) and finally, the pro‐inflammatory and anti‐inflammatory profile identity was determined by CD68 (1:1000, 137,025, Alexa Fluor 700 BioLegend) and CD206 (1:1000, 141,717, Brilliant Violet 421, BioLegend) antibodies, respectively.

### 4.6 IGF2‐Reprogrammed Macrophages Treatment in ASO Mice

Breeding pairs of the Thy‐1 α‐synuclein overexpressing (ASO) line of transgenic mice were purchased from the Jackson Laboratories. Mice were housed in a temperature and humidity‐controlled room under a 12 h light–dark cycle. Mice were given ad libitum access to food and water. Male ASO mice were crossed with female C57BL/6 WT mice, and the resultant male WT and ASO mice were used for all the experiments performed in this study. For proper comparison, all biochemical, histological, and behavioral analyses were carried out on groups of littermates of the same breeding generation. Animal care and experimental protocols were conducted according to procedures approved by the Bioethical Committee of the Center for Integrative Biology, University Mayor (07–2017).

To determine the effect of IGF2‐macrophages (MIGF2) on the inflammatory response associated with PD, intravenous transfer of IGF2‐macrophages (2 × 10^5^ cells) in ASO and WT mice was performed. The macrophages intravenous transfer has been previously described in the PD preclinical model, and its distribution is mainly in inflammatory tissue (Chen et al. 2018; Haney et al. 2020; Zhao et al. 2014). IGF2‐macrophages were intravenously transferred to ASO mice at the presymptomatic, early symptomatic, and late‐symptomatic stages of PD progression (3, 14, and 23 months old, respectively) weekly for 4 weeks. Four different experimental groups (5–8 mice per group) were generated.

1. WT mice injected with macrophage control (MC).
2. WT mice injected with IGF2‐reprogrammed macrophages (MIGF2).
3. ASO mice injected with macrophage control (MC).
4. ASO mice injected with IGF2‐reprogrammed macrophage (MIGF2).

During treatment, animal health was monitored every day. Body weight was recorded every week starting at the first week of treatment. Motor performance was evaluated by the Beam traversal test, Clasping test, and Adhesive removal test.

### 4.7 Motor Test Assessment

The beam traversal test, which is particularly useful for detecting subtle deficits in motor skills and balance, was previously described (Fleming et al. 2004, 2006). Briefly, animals were trained to traverse the length of a Plexiglas beam consisting of four sections (25 cm each, 1 m total length) of different widths (3.5 cm to 0.5 cm by 1 cm increments) with support ledges attached along each side leading to the food. After 2 days of training, a mesh grid (1 cm^2^) of corresponding width was placed over the beam surface, leaving approximately a 1 cm space between the grid and the beam surface. The day after, animals were videotaped while traversing the grid‐surfaced beam for two trials at the basal state and after treatment with MC or MIGF2. Videotapes were viewed and rated in slow motion for step errors, considering the number of steps made and the time to traverse across by each animal by an investigator blind to the mouse genotype and treatment. Scores were calculated across both trials and averaged for each mouse.

The clasping test is a functional motor test that quantifies deficits in corticospinal function as a previously described (Lalonde and Strazielle 2011). Briefly, mice were held by the tail for 5–10 s. During tail suspension, limb‐clasping behavior was recorded and manually evaluated with the following standards: 0—no limb clasping, normal escape extension; 1—either hind limbs or forelimbs incomplete splay and loss of mobility; 2—either hind limbs or forelimbs clasping together and loss of mobility; 3—single side hind limb clasping together and loss of mobility; 4—both hind limbs clasping together and loss of mobility. This test was performed at the basal state and after the treatment with MC or MIGF2.

The adhesive Removal test is an assay to determine the sensorimotor function in mice as previously described (Fleming et al. 2004). Briefly, small adhesive stimuli (adhesive‐backed labels, one‐quarter inch round) were placed on the snout of the mouse, and the time to make contact and remove the stimulus was recorded. All testing was performed in the animal's home cage, and cage mates were temporarily removed during testing because they could interfere with stimulus removal. If the animal did not remove the stimulus within 60 s, the experimenter removed it, and the trial for the next mouse was initiated. This test was performed at the basal state and after treatment with MC or MIGF2.

### 4.8 Immunohistochemistry Analysis in ASO Mice

WT and ASO mice were euthanized by isoflurane overdose. The brains were then isolated and immediately fixed with 4% PFA overnight at 4°C. The next day, the samples were embedded in paraffin and cut at 10 μm with a microtome. Then, immunochemistry was performed; firstly, antigen retrieval was performed with 1 mM citrate buffer pH 6.0 for 45 min at streamer. For nonspecific reaction blocking was used 5% Bovine serum albumin, 1% donkey normal serum, and 0.5% horse normal serum in PBS1x solution were used for 2 h. Primary antibodies were incubated overnight at 4°C; subsequently, the polymer kit detection system (MP‐7800, Vector Laboratories) was used for 1 h. To determine the IBA1 positive cells in *the substantia nigra* brain region using an anti‐IBA1 antibody (1:1000, 019–19,741, Wako Chemicals). The presence of α‐syn inclusion in the substantia nigra brain region was measured using anti‐phosphor‐α‐syn (1:500, 825701, BioLegend).

In case IBA1 positive cells, the number and morphology were analyzed as described previously (Jin and Yamashita 2016; Kreutzberg 1996; Wittekindt et al. 2022), considering soma size and shape as well as the number of branches, to categorize the microglia as either “reactive/active” or “resting”. The amount of phosphor‐α‐syn was determined by integrated density using ImageJ.

### 4.9 Immunofluorescence in Macrophages Primary Culture

BMDMs from WT and ASO mice were immediately fixed with 4% PFA for 15 min at room temperature (RT) and washed briefly. Then, immunofluorescence was performed; firstly, permeabilization was realized with 0.5% BSA, 0.5% NP‐40 in PBS1x for 15 min. Next, for nonspecific blocking was used a 0.5% Bovine serum albumin and 10% SFB in PBS1x solution were used for 15 min. Anti‐TLR4 (1:1000, PA5‐23124, ThermoFischer Scientific) and MHCII (1:500, NBP2‐21789, Novus Biological LLC) antibodies were incubated overnight at 4°C; subsequently, the samples were incubated with secondary antibodies with DAPI (1:10000, 28718‐90‐3, BioLegend) and mounted with Dako fluorescent mounting medium (C0563, Dako). Representative images for each experiment were obtained from Leica DMI8 epifluorescence microscopy (Leica microsystems) and then the protein expression of TLR4 and MHCII was determined, respectively, by integrated density using ImageJ.

### 4.10 Western Blot Analysis

For western blot analysis, brain tissues were homogenized in RIPA buffer (20 mM Tris pH 8.0, 150 mM NaCl, 0.1% SDS, 0.5% Triton X‐100) containing protease and phosphatase inhibitors (11697498001, Roche, Merck). After sonication, the protein concentration was determined in all experiments by micro‐BCA assay (Pierce), and 15–25 μg of total protein was loaded onto 4%–15% SDS‐PAGE mini gels (4,561083, Bio‐Rad Laboratories) prior to transfer onto 0.25 μm PVDF membranes. Membranes were blocked using Tris buffered saline (TBS), 0.1% Tween‐20 (TBST) containing 5% milk for 60 min at room temperature and then probed overnight with primary antibodies in TBS, 0.02% Tween‐20 (TBST) containing 5% skimmed milk. The following primary antibodies and dilutions were used: anti‐alpha‐synuclein (1: 1000, 610787, BD Transduction), anti‐beta‐actin‐HRP (1:5000, sc‐47778, Santa Cruz Biotechnology). Bound antibodies were detected with peroxidase‐coupled secondary antibodies incubated for 2 h at room temperature and the ECL system (34577, ThermoFische Scientific). The amount of total α‐syn and β‐actin levels was determined by integrated density using ImageJ.

### 4.11 Lactate Dehydrogenase (LDH) Technique for Cell Viability

Lactate dehydrogenase (LDH) activity was measured according to manufacturer protocol (88953, ThermoFischer Scientific). Briefly, in a 96‐well plate, 50 μL of each sample medium was added, followed by an additional 50 μL of the reaction mixture and mixed gently. The plate was then incubated at room temperature for 30 min, protected from light. Finally, 50 μL of Stop Solution was added to each sample, and the LDH activity was measured at the absorbance at 490 and 680 nm.

### 4.12 Mitochondrial Activity Measurement

Mitochondrial superoxide radical production was analyzed by flow cytometry using a dihydropyridine derivative probe 5 μM of MitoSOX (Martin‐Montanez et al. 2021). Mitochondrial function was measured as variation in OCR by Seahorse technology, mitochondrial potential (mΔΨ) by flow cytometry using JC1 probe, and COX activity by spectrophotometric methods (Martin‐Montanez et al. 2017).

### 4.13 Statistical Analysis

Analysis of macrophage inflammatory profile, mRNA expression levels of PBMCs from humans and mice using the Mann–Whitney test after the Shapiro–Wilk test confirmation of non‐parametric samples. N indicates the number of subjects. Also, the analysis of motor performance from WT and ASO mice was determined using the one‐way ANOVA post‐test Tukey's. N indicates the number of mice. Inflammatory profile, mRNA expression levels and protein levels in macrophage cell culture, and mitochondria activity in SN4741 were analyzed using ordinary one‐way ANOVA post‐test Tukey's (parametric data). N indicates the number of biological replicates.

All graph's error bars represent SEM (standard error for the media). GraphPad Prism software 9.0 was used for statistical analysis.

## Author Contributions

Conceptualization: F.G., R.L.V., D.S., and R.P. Methodology: F.G., R.P., and R.L.V. Investigation: F.G., R.L.V., T.J.H., D.S., C.J., V.U., B.C., S.C., R.P., E.M.‐M., N.V., and M.G.‐F. Resources: R.L.V., R.D., J.T., E.M.‐M., M.G.F., C.A., P.C.‐C, and R.P. Writing – original draft: R.L.V., F.G., T.J.H., and R.P. Project administration: R.L.V. Funding acquisition: R.L.V., E.M.‐M., and M.G.‐F. Dr. Felipe Grunenwald and Mr. Tomás J. Huerta have contributed equally to this work and share the first authorship.

## Conflicts of Interest

The authors declare no conflicts of interest.

## Supporting information


**Figure S1.** Macrophage identity of differentiated Bone Marrow Monocytes. Flow cytometric analysis of mouse cells (ASO and WT mice) isolated from bone marrow. (A) Lymphoid cell selection with CD45+. (B) Single cells were gated using FSC‐W and FSC‐H gating. (C) Cell death detection using zombie aqua stain. (D) Macrophages were selected using CD11B^+^ and F4/80^+^. For all experiments, the mean and standard error are represented for 5 samples per condition.
**Figure S2.** IGF2 treatment decreases the expression of TLR4 and MHCII in macrophages exposed to α‐syn. Analysis of TLR4 and MHCII in primary culture of macrophages co‐treatment with αsyn‐PFF and rIGF2. (A) TLR4 and MHCII immunodetection in primary cultures of macrophages treated with αsyn‐PFF and/or rIGF2. (B) Determination of integrated density of TLR4 in macrophages primary culture. (C) Determination of integrated density of MHCII in macrophages primary culture. Scale bar 25 μm. In all quantifications, statistically significant differences were detected by ANOVA post‐test Tukey’s (****: *p* < 0.0001; ***: *p* < 0.001; **: *p* < 0.01; *: *p* < 0.05). LPS was used as positive control of inflammatory response. For all experiments, the mean and standard error are represented for 3 samples per condition.
**Figure S3.** MIGF2 treatment decreases α‐syn levels in PD preclinical mode at 23 months. Western Blot analysis was performed in the Substantia Nigra (SN) brain region from 23‐month‐old ASO, or WT mice treated with macrophages reprogrammed with IGF2 (MIGF2) or macrophages reprogrammed with PBS (MC). (A) Total α‐syn immunodetection in the SN brain region from ASO or WT treated with MC or MIGF2. (B) Quantification of total α‐syn levels in SN brain region and normalized to β‐actin levels as a loading control. In all quantifications, statistically significant differences were detected by ANOVA post‐test Tukey’s (**p < 0.01; *p < 0.05). For all experiments, the mean and standard error are represented for 5–6 samples per condition.
**Figure S4.** IGF2‐reprogrammed macrophages reduce TLR4 signaling in PBMC from PD model at 23 and 14 months. (A–C) Total mRNA was extracted from freshly isolated peripheral blood mononuclear cells (PBMCs) from the ASO, and WT mice treated. (A) Cas‐1 mRNA levels were quantified and normalized to β‐actin levels at 23 months. (B) mRNA levels of Nlrp3 were quantified and normalized to β‐actin levels at 23 months. (C) Cas‐1 mRNA levels were quantified and normalized to β‐actin levels at 14 months. (D) mRNA levels of Nlrp3 were quantified and normalized to β‐actin levels at 14 months. In all quantifications, statistically significant differences were detected through one‐way ANOVA post‐test Tukey’s (** *p* < 0.01; * *p* < 0.05). For all experiments, the mean and standard error are represented for 7–8 mice per condition.
**Figure S5.** IGF2‐reprogrammed macrophages reduce inflammatory response in preclinical PD model at 14 months. (A–C) Flow cytometric analysis of freshly isolated peripheral blood mononuclear cells (PBMCs) from 23‐month‐old ASO or WT mice treated with MIGF2 or MC. (A) Cell selection parameters of macrophage cells (macrophage F4/80^+^ CD11b^+^) staining with surface marker CD206 and CD68. (B) Percentages of CD206−/CD68+ macrophages population. (C) Percentages of CD206+/CD68− macrophages population. In all quantifications, statistically significant differences were detected through one‐way ANOVA post‐test Tukey’s (***p < 0.001; **p < 0.01; *p < 0.05). For all experiments, the mean and standard error are represented for 7–8 mice per condition.
**Figure S6.** MIGF2 treatment modified the expression of proinflammatory cytokines in PD preclinical model at 3 months. Cytokines expression and macrophage inflammatory profile was evaluated in 3‐month‐old ASO or WT mice with macrophages reprogrammed with IGF2 (MIGF2) or macrophages reprogrammed with PBS (MC). (A–C) Total mRNA was extracted from freshly isolated peripheral blood mononuclear cells (PBMCs) from the ASO and WT mice. (A) mRNA levels of mIL‐1β were quantified and normalized to β‐actin levels. (B) mRNA levels of mTNF‐α were quantified and normalized to β‐actin levels. (C) mRNA levels of mIL‐10 were quantified and normalized to β‐actin levels. (D–F) Flow cytometric analysis of isolated peripheral blood mononuclear cells (PBMCs) from 3‐month‐old ASO or WT mice treated with MIGF2 or MC. (D) Cell selection parameters of macrophage cells (macrophage F4/80+ CD11b+) staining with surface marker CD206 and CD68. (E) Percentages of CD206‐/CD68+ macrophages population. (F). Percentages of CD206+/CD68‐ macrophages population. In all quantifications, statistically significant differences were detected through one‐way ANOVA post‐test Tukey’s (***p < 0.001; **p < 0.01; *p < 0.05). For all experiments, the mean and standard error are represented for 5–6 mice per condition.

## Data Availability

Data is available on request from authors.
