# Supplementary figures and images for: IGF2‐Reprogrammed Macrophages Ameliorate the Inflammatory Response and Protect Against the Neuroinflammatory Process in Parkinson's Disease Models
Source: Aging Cell. 2025 Mar 27;24(6):e70020. doi: 10.1111/acel.70020 (PMC12151900; doi:10.1111/acel.70020)

Supplemental material

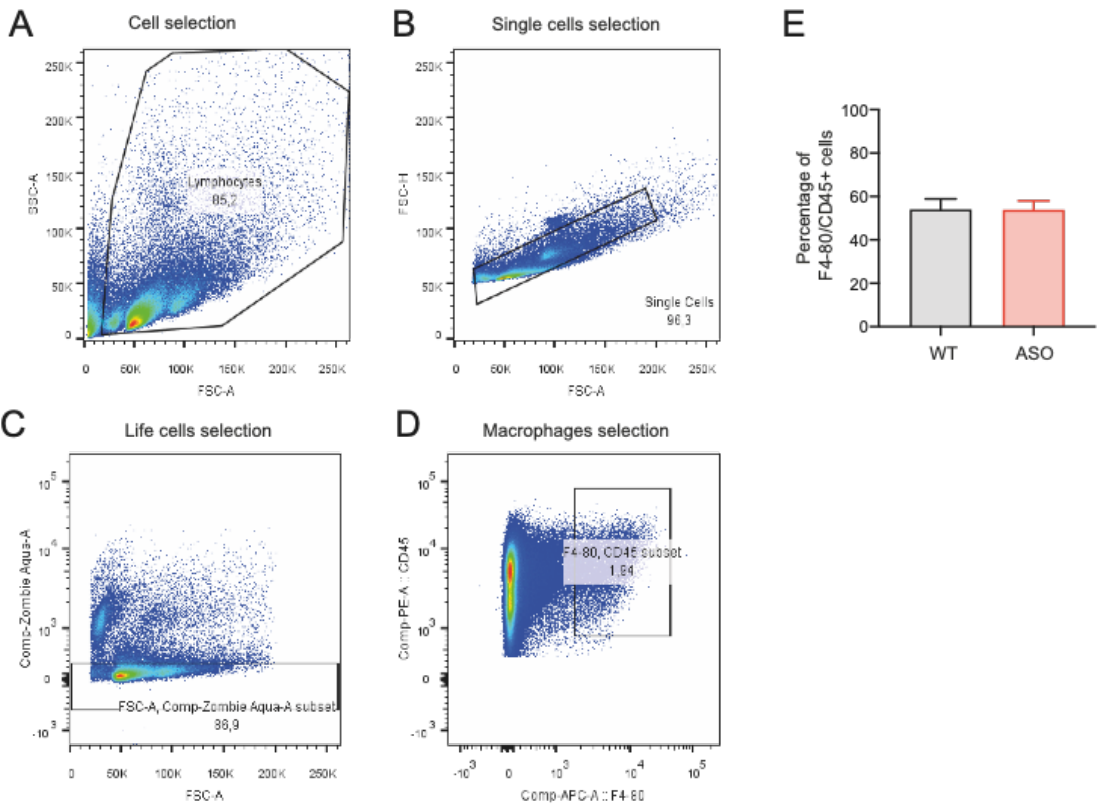

Figure S1

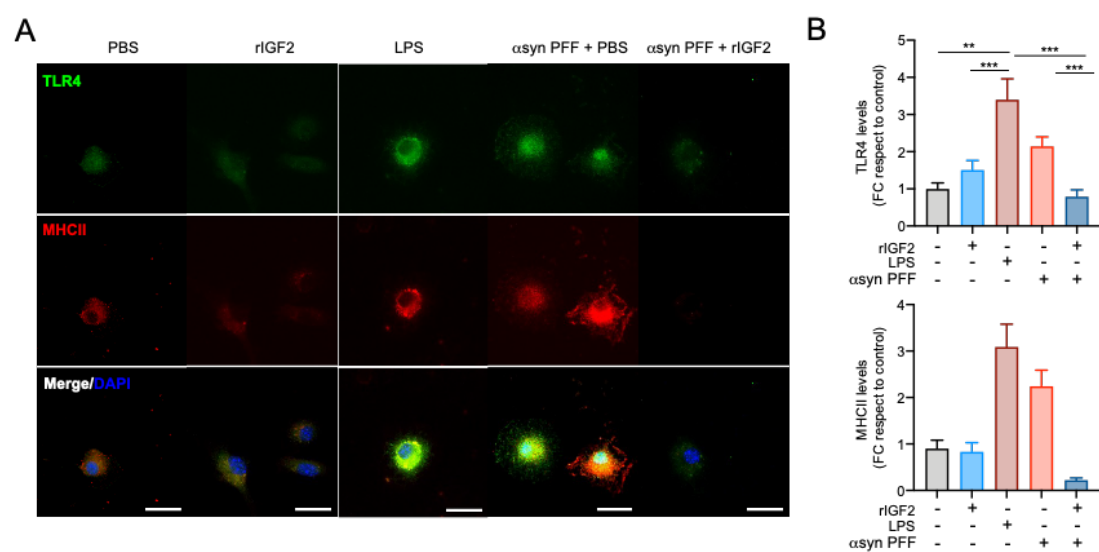

Figure S2

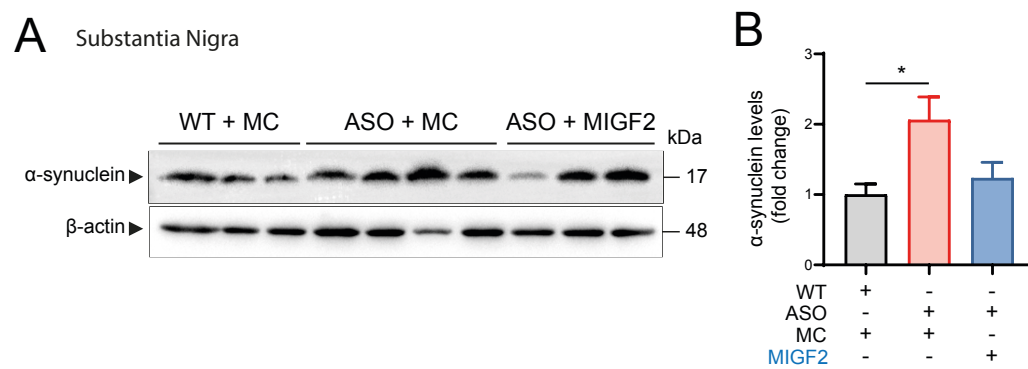

Figure S3

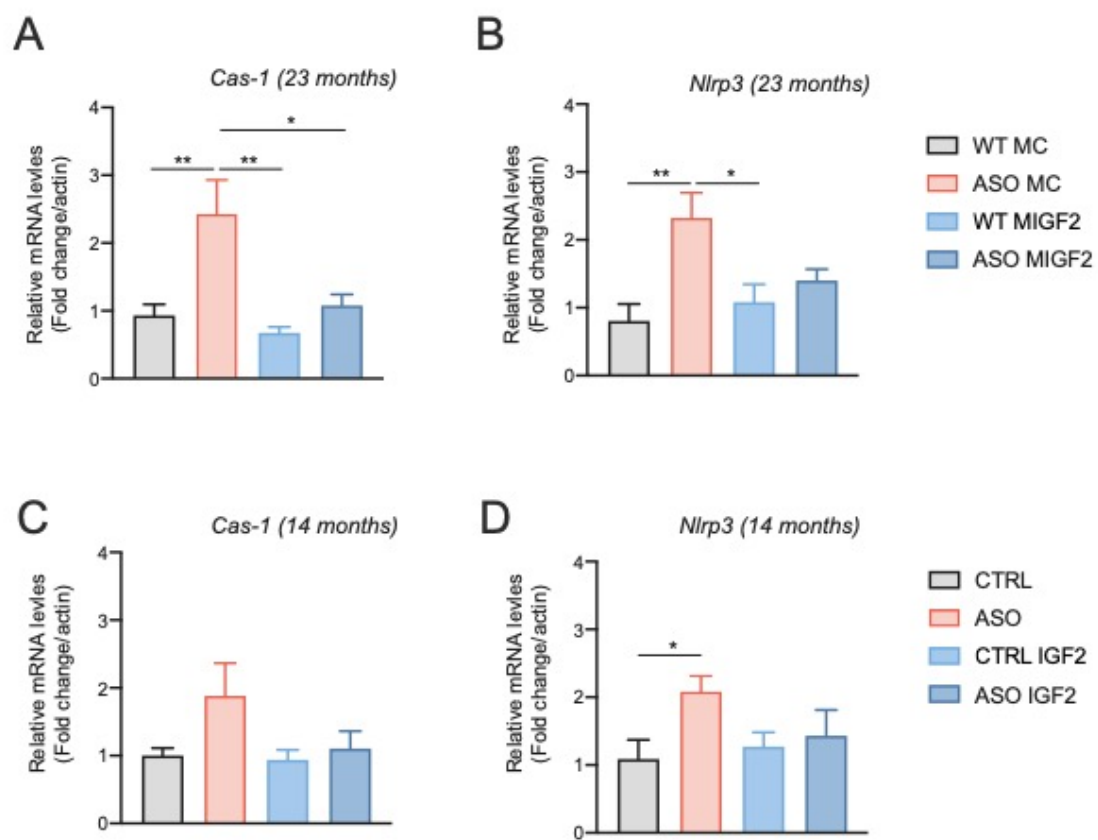

Figure S4

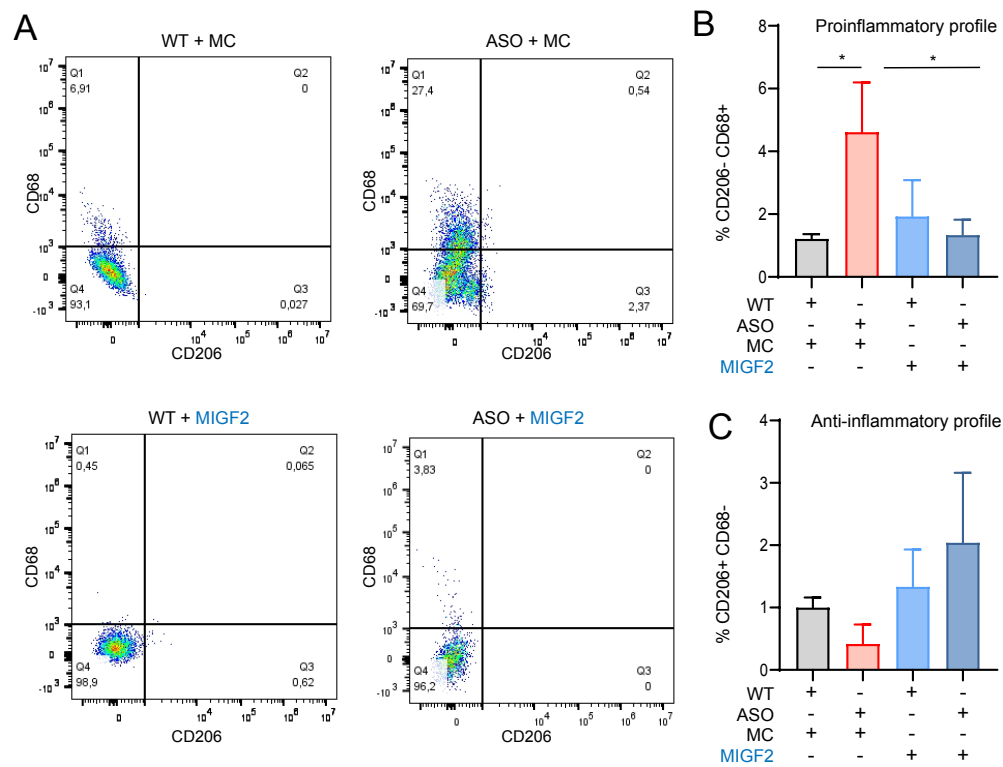

Figure S5

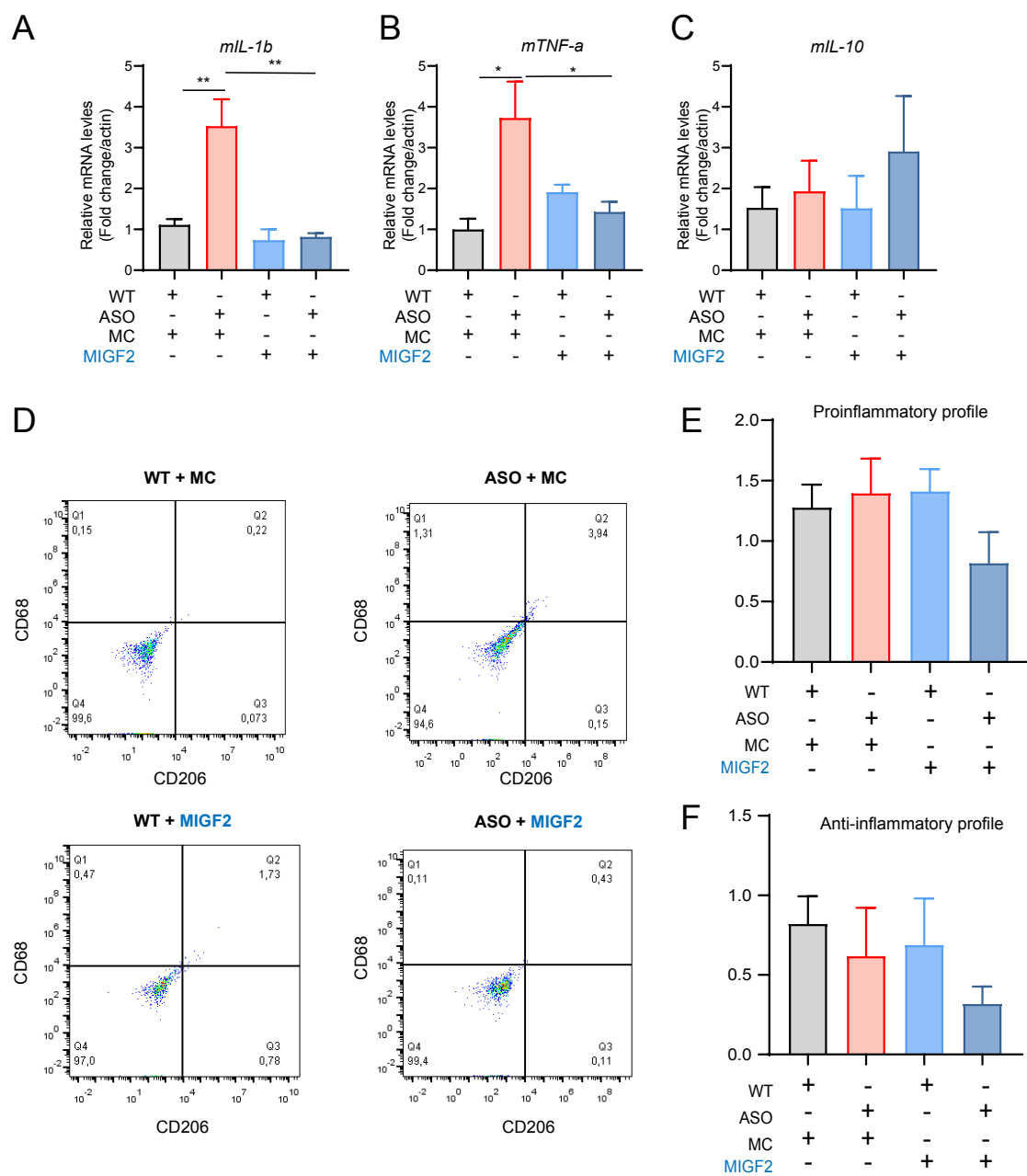

Figure S6

Supplement: Supplementary file 1 — Figure S1. Macrophage identity of differentiated Bone Marrow Monocytes. Flow cytometric analysis of mouse cells (ASO and WT mice) isolated from bone marrow. (A) Lymphoid cell selection with CD45+. (B) Single cells were gated using FSC‐W and FSC‐H gating. (C) Cell death detection using zombie aqua stain. (D) Macrophages were selected using CD11B+ and F4/80+. For all experiments, the mean and standard error are represented for 5 samples per condition. Figure S2. IGF2 treatment decreases the expression of TLR4 and MHCII in macrophages exposed to α‐syn. Analysis of TLR4 and MHCII in primary culture of macrophages co‐treatment with αsyn‐PFF and rIGF2. (A) TLR4 and MHCII immunodetection in primary cultures of macrophages treated with αsyn‐PFF and/or rIGF2. (B) Determination of integrated density of TLR4 in macrophages primary culture. (C) Determination of integrated density of MHCII in macrophages primary culture. Scale bar 25 μm. In all quantifications, statistically significant differences were detected by ANOVA post‐test Tukey’s (****: p < 0.0001; ***: p < 0.001; **: p < 0.01; *: p < 0.05). LPS was used as positive control of inflammatory response. For all experiments, the mean and standard error are represented for 3 samples per condition. Figure S3. MIGF2 treatment decreases α‐syn levels in PD preclinical mode at 23 months. Western Blot analysis was performed in the Substantia Nigra (SN) brain region from 23‐month‐old ASO, or WT mice treated with macrophages reprogrammed with IGF2 (MIGF2) or macrophages reprogrammed with PBS (MC). (A) Total α‐syn immunodetection in the SN brain region from ASO or WT treated with MC or MIGF2. (B) Quantification of total α‐syn levels in SN brain region and normalized to β‐actin levels as a loading control. In all quantifications, statistically significant differences were detected by ANOVA post‐test Tukey’s (**p < 0.01; *p < 0.05). For all experiments, the mean and standard error are represented for 5–6 samples per condition. Figure S4 [file ACEL-24-e70020-s001.pdf]
